# RNA processing kinase inhibitors and epigenetic inhibitors in combination with oncology drugs or investigational agents in multi-cell type patient-derived tumor cell line spheroids

**DOI:** 10.1007/s00280-025-04800-w

**Published:** 2025-09-12

**Authors:** Beverly A. Teicher, Thomas S. Dexheimer, Thomas Silvers, Nathan P. Coussens, Eric Jones, Steven D. Gore, Mark Kunkel, James H. Doroshow

**Affiliations:** 1 Applied and Developmental Research Directorate, Frederick National Laboratory for Cancer Research, Frederick, MD 21702 USA; 2 Division of Cancer Treatment and Diagnosis, National Cancer Institute, Bethesda, MD 20892 USA; 3 Molecular Pharmacology Branch, National Cancer Institute, RM 4-W602, MSC 9735, 9609 Medical Center Drive, Bethesda, MD 20892 USA

**Keywords:** cirtuvivint, iadademstat, CC-671, spheroids, eltanexor, MRTX-1133

## Abstract

**Purpose:**

The alternative splicing of mRNA precursors allows one gene to yield multiple proteins with distinct functions. CDC-like kinases serve as pivotal regulators of alternative splicing. Control of protein expression also occurs at the level of DNA through histone methylation and demethylation. We investigated the activity of two CLK inhibitors, cirtuvivint and CC-671, and the LSD1 inhibitor iadademstat alone and in combination with anticancer drugs or investigational agents.

**Methods:**

Well-characterized patient-derived cancer cell lines from the PDMR (https://pdmr.cancer.gov/models/database.htm) were used along with standard human cancer cell lines. Multi-cell type-tumor spheroids were grown from a ratio of 6:2.5:1.5 malignant cells, endothelial cells, and mesenchymal stem cells. Following three days of growth, the spheroids were exposed to the single agents or combinations at concentrations up to the clinical C_max_ value for each agent, if known. After seven days of exposure, cell viability was assessed using the CellTiter-Glo 3D assay and spheroid volume was assessed by bright field imaging.

**Results:**

Several of the targeted oncology drugs exhibited additive and greater-than-additive cytotoxicity when combined with a CLK inhibitor, or the LSD1 inhibitor. These agents included the XPO1 inhibitor, eltanexor, and the KRAS G12D specific inhibitor MRTX-1133 which had activity in tumor lines harboring the KRAS G12D mutation. LSD1 inhibition was effective with ubiquitin proteasome pathway inhibitors. Conclusion: These findings may provide guidance for development of clinical trial combination regimens including cirtuvivint, CC-671 or iadademstat. Full data sets are available on PubChem.

## Introduction

The spliceosome, a critical intracellular organelle, is multi-megadalton complex composed of over 100 proteins including 5 small nuclear ribonucleoproteins. Cancer cells often have cancer type-specific RNA splicing alterations. Most multi-exon human genes undergo alternative splicing, allowing multiple mature mRNAs to be derived from a single gene [1, 2]. Thus, 25,000 genes can code for well over 100,000 proteins. Pre-mRNA splicing, the process of removing introns from precursor messenger RNA, is critical in the post-transcriptional regulation of gene expression [3]. Serine/arginine (SR) family proteins control the patterns of alternative splicing in pre-mRNA and enhance splicing from nearby splice sites by interacting with exonic and intronic splicing enhancer sequences in pre-mRNA [3]. SR-proteins require phosphorylation by SR protein kinases (SRPKs), protein kinase B (PKB/AKT), NIMA-related kinase2 (NEK2), PRP4 kinase (PRP4K) dual-specificity tyrosine phosphorylation-regulated kinase 1 A (DYRK1A), cAMP-dependent protein kinase (PKA) or by the CDC-like kinases (CLKs) to be active [4]. The CLKs regulate transcript RNA splicing through SR protein phosphorylation [5]. Cirtuvivint is a pan CDC-like kinase (CLK1-4) and dual specificity tyrosine kinase (DYRK1-4) inhibitor which targets mRNA splicing and the Wnt pathway which is in clinical trial [6]. Cirtuvivint exposure disrupts spliceosome activity and decreases production of Wnt signal pathway splicing variants. A Phase I clinical trial of cirtuvivint by oral administration, assessing dose escalation is currently active but not recruiting (NCT03355066) and the combination of cirtuvivint with ASTX727, a combination of decitabine and cedazuridine, in patients with acute myeloid leukemia or myelodysplastic syndromes is actively accruing patients (NCT06484062) [5]. CC-671, a CLK/TTK inhibitor has an inhibitory repertoire similar to cirtuvivint. TTK, also called Monopolar spindle 1 (Mps1), is a dual serine/threonine kinase that regulates the spindle assembly checkpoint, controlling cellular progression through mitosis [7, 8]. CC-671 had in vivo activity in human tumor xenografts due to dual inhibition of CLK2/TTK [4].

Lysine-specific demethylase 1α (LSD1) encoded by the KDM1A gene, is a lysine demethylase which has recurrent mutations, translocations, and somatic copy number gains or losses in human tumors [9, 10]. LSD1/KDM1A removes mono- and dimethyl groups of histone H3 lysine-4 (H3K4), lysine-9 (H3K9) as well as non-histone substrates [11, 12]. LSD1 interacts with other chromatin regulators including histone deacetylases (HDAC)−1, 2 and 3, and DNA methyltransferase 1 (DNMT1). LSD1 is a component of a multi-subunit complex causing transcription activation or repression [13]. LSD1 non-histone substrates are associated with the regulation of cell cycle progression and apoptosis. LSD1/KDM1A expression is associated with poor prognosis in prostate, breast, lung, bladder, colorectal cancer and neuroblastoma. Iadademstat is a potent LSD1 inhibitor with an IC_50_ of 18 nM and > 1000-fold higher selectivity towards LSD1 compared to related FAD-dependent aminoxidases [14, 15]. In AML models, LSD1 inhibition did not alter genome methylation but did increase me_2_H3K4 at LSD1 target genes [16–18]. In a small cell lung cancer xenograft, treatment with iadademstat resulted in NOTCH activation and ASCL1 suppression decreasing neuroendocrine properties [19].

With the complexity of cancer genomic alterations, single targeted drugs are usually not sufficient to impact malignant disease, and combinations of targeted drugs are necessary for effective treatment. The current study was undertaken to explore the activity of RNA processing and epigenetic inhibitors in combination with other targeted agents and standard-of-care drugs in a multi-cell type (mct)-spheroid model from patient-derived tumor cell lines of the PDMR (https://pdmr.cancer.gov/) or standard tumor cell lines and stromal cells. The full data sets are available at the PubChem files listed on Table 4.

## Materials and methods

**Compounds**. The drugs and investigational agents: cirtuvivint (SM08502; NSC835563), CC-671 (NSC850746), iadademstat (NSC806812), vemurafenib (NSC761431), tapotoclax (NSC804041), sotorasib (NSC818433), adagrasib (NSC831453), MRTX-1133 (NSC836407), BAY2416964 (NSC825713), copanlisib (NSC816437), inavolisib (NSC800729), abemaciclib (NSC768073), osimertinib (NSC779217), entinostat (NSC756642), eltanexor (KPT-8602, NSC794443), venetoclax (NSC766270), ceralasertib (NSC777638), bortezomib (NSC756655), olaparib (NSC753686), talazoparib (NSC767125), adavosertib (NSC754677), alisertib (NSC759677), ART-558 (NSC835418), aza-T-dCyd (NSC777586), AZD-1390 (NSC803789), belinostat (NSC758774), camonsertib (NSC841442), CPI-455 (NSC825282), decitabine (NSC127716), elimusertib (NSC800525), ixazomib (NSC758254), KSQ-4279 (NSC840948), panobinostat (NSC761190), peposertib (NSC802822), pevonedistat (NSC761192), R306465 (NSC773264), selinexor (NSC780203), TAK-243 (NSC785004), tazemetostat (NSC777109), topotecan (NSC609699), TP-3654 (NSC805149), eribulin (NSC707389), etoposide (NSC141540), carboplatin (NSC241240), cisplatin (NSC119875), gemcitabine (NSC613327), 5-fluorouracil (NSC19893), SN-38 (NSC673596), oxaliplatin (NSC266046), paclitaxel (NSC125973), and doxorubicin (NSC123127), were obtained from the National Cancer Institute (NCI) Developmental Therapeutics Program Chemical Repository [20]. The FDA-approved anticancer drug set is available from the Developmental Therapeutics Program at https://dtp.cancer.gov/organization/dscb/obtaining/available plates.htm. The drugs and investigational agents used were demonstrated to be > 95% pure by proton nuclear magnetic resonance and liquid chromatography/mass spectrometry. The stock solutions were prepared in dimethyl sulfoxide (DMSO, Sigma-Aldrich, St. Louis, MO, cat. D2650), except for the platinum complexes which were prepared in saline (Quality Biological, Gaithersburg, MD, cat. 114-055-101), at 800-fold the tested concentration and stored at −70 °C prior to their use. All drugs and investigational agents were tested over a range starting from a high concentration at or near the clinical C_max_ and decreasing in half-log increments. If the clinical C_max_ for an agent had not been determined, the highest concentration tested was 10 µM (Table 1).

**Table 1:** Drugs and investigational agents for Pidnarulex (CX-5461), APTO-253(LOR-253), BRACO-19, iadademstat, cirtuvivint and CC-671 combination mct-spheroid screens

Drug or Invest Ag	NSC #	MW	Clin Cmax (10 μm default)	Target
Cirtuvivint	835563	427.5	3 µM	CLKs/DYRKs
CC-671	805746	512.6	10 µM	CLK2/TTK
Iadademestat	806812	230.35	10 µM	LSD1 (KDM1A)
5-Fluorouracil	19893	130.1	426 µM	Thymidylate synthase
Abemaciclib	763073	506	0.59 µM	CDK4/6
Adagrasib	831453	604.12	3 µM	KRAS G12C
Adavosertib (MK-1775)	754352	500.6	10 µM	WEE-1
Alisertib	759677	519	10 µM	Aurora Kinase
ART-558	835418	418.4	10 µM	POLQ
Aza-T-dCyd	777586	244.27	0.3 µM	DNMT1
AZD-1390	803789	477.6	10 µM	ATM
BAY2416964	825713	378.8	10 µM	AhR inhibitor
Belinostat	758774	318	134 µM	HDAC
Bortezomib	756655	394	0.312 µM	Proteasome
Camonsertib	841442	410.5	10 µM	ATR
Carboplatin	241240	371.3	135 µM	DNA crosslinking
Ceralasertib	780249	412.5	10 µM	ATR
Cisplatin	119875	300	14.4 µM	DNA
Copanlisib	816437	480.53	0.964 µM	AKT α/δ
CPI-455	825282	314.77	10 µM	KDM5
Dacitabine	127716	228	0.323 µM	DNMT1
Doxorubicin	123127	544	6.73 µM	TopII/DNA intercalator
Elimusertib	800525	375.4	10 µM	ATR
Eltanexor	794443	428.3	10 µM	XPO1
Entinostat	756642	376.4	10 µM	HDAC
Eribulin	707389	730	0.508 µM	Tubulin
Etoposide	141540	588.6	33.4 µM	TopII inhibitor
Gemcitabine	613327	263	89.3 µM	Antimetabolite/
Inavolisib	800729	407.4	10 µM	PIK3CA, PI3Ka
Ixazomib	758254	517	0.118 µM	Proteasome
KSQ-4279 (R07623066)	840948	534.54	10 µM	USP1
MRTX-1133	836407	600.63	10 µM	KRAS G12D
Olaparib	753686	434	13.1 µM	PARP
Osimertinib	779217	500	0.126 µM	EGFR
Oxaliplatin	266046	397	4.96 µM	DNA crosslinker
Paclitaxel	125973	854	4.27 µM	Tubulin stabilizer
Panobinostat	761190	349	0.082 µM	HDAC
Peposertib	802822	481.1	10 µM	DNA-PK
Pevonedistat	761192	443.5	10 µM	NAE (NEDD8)
R306465	773264	413.5	10 µM	HDAC
Selinexor	780203	443.3	1.53 µM	CRM1
SN-38	673596	392.4	–	TopI inhibitor
Sotorasib	818433	561	10 µM	KRAS G12C
TAK-243	785004	519.5	0.3 µM	UAE
Talazoparib	767125	380.4	0.043 µM	PARP
Tapotoclax	804041	613.2	10 µM	MCL-1
Tazemetostat	777109	572.75	1.45 µM	EZH2
Topotecan	609699	421	0.015 µM	TopI inhibitor
TP-3654	805149	418.5	10 µM	PIM-1
Vemurafenib	761431	490	127 µM	BRAF V600E
Venetoclax	766270	468	4.48 µM	BCL-2

**Cell Lines**. The patient-derived cancer (PDC) cell lines include 16 colon adenocarcinoma lines: 186277-243-T-J2-PDC, 188146-221-R-J1-PDC, 282377-053-R-J1-PDC, 381356-305-R-J1-PDC, 435261-313-R-J1-PDC, 439559-082-T-J2-PDC, 519858-162-T-J1-PDC, 616215-338-R-J1-PDC, 695427-040-R-J1-PDC, 233499-124-R-J2-PDC, 817829-284-R-J1-PDC, 825966-067-R-J1-PDC, 857933-349-R-J2-PDC, 996289-038-R-J1-PDC, 997537-175-T-J1-PDC, and 947758-054-R-J2-PDC; 16 pancreatic carcinoma lines: 217524-143-R1-J4-PDC, 227483-062-R1-J1-PDC, 242566-281-R-J2-PDC, 292921-168-R-J2-PDC, 323965-272-R-J2-PDC, 377384-186-R-J1-PDC, 422866-222-R5-J1-PDC, 454973-116-R2-J3-PDC, 485176-168-R4-J1-PDC, 485368-065-R4-J2-PDC, 521955-158-R2-J5-PDC, 777334-354-R1-J3-PDC, 885724-159-R-J1-PDC, 966289-007-R4-J1-PDC, K24384-001-R-PDC, and 496974-208-R-J2-PDC; 6 bladder carcinoma lines: 168753-222-R-J1-PDC, 324938-238-R-J1-PDC, 565232-114-T-J1-PDC, 648629-189-R-J1-PDC, 883617-216-R-PDC, and 855422-203-R-J1-PDC; 7 endometrial carcinomas: 379773-124-R-J2-PDC, 598228-144-R-J1-PDC, 633275-114-R-J1-PDC, 636577-100-R-J1-PDC, 922993-354-T-J2-PDC, and 876862-298-R-J1-PDC; 3 head & neck squamous carcinoma: 328373-195-R-J1-PDC, 628569-122-R-J1-PDC, and 874868-142-R-J2-PDC; 4 melanoma: 156681-154-R-J1-PDC, 276233-004-R-J1-PDC, 299254-011-R-J1-PDC, and 876135-273-R-J2-PDC; 3 NSCLC: 349418-098-R-PDC, 653999-131-R-J2-PDC, and LG0703-F948-PDC; 2 breast carcinoma: 171881-019-R-J1-PDC and 885512-296-R-J2-PDC; 2 MPNST: 317291-083-R-J1-PDC and 596521-263-R-J1-PDC; 1 ovarian carcinoma: 556581-035-R-J1-PDC; 1 renal cell carcinoma: 743489-274-T-PDC; and 1 Merkel cell tumor: 138582-337-R-J1-PDC, were obtained from the NCI Patient-Derived Models Repository (PDMR, https://pdmr.cancer.gov/) (Table 2). In addition, several standard human tumor cell lines were used including: 786-0 and A498 renal cell carcinoma, IGROV1, NCI/ADR-RES and OVCAR-5 ovarian carcinoma; MDA-MB-231, MDA-MB-468, and SUM149PT TNBC; NCI-H1876, NCI-H196, NCI-H211 and SW 1271 SCLC; A375 melanoma and K562 leukemia were obtained from the NCI Tumor Repository (Table 2). Pooled donor human umbilical vein endothelial cells (HUVEC, Lonza, cat. CC-2519) and human mesenchymal stem cells (hMSC, Lonza, cat. PT-2501) were purchased from Lonza (Walkersville, MD).

**Table 2:** Tumor cell lines tested as complex spheroids (including endothelial cells and mesenchymal stem cells) for response to cirtuvivint, CC-671 or Iadademstat alone and in simultaneous combination with the compounds in Table 1

Cell Line	Disease	Key Mutations
138582-337-R-J1-PDC	Merkel Cell	PMS2, PTEN
156681-154-R-J1-PDC	Melanoma	SF3B1 R196Q; BRAF V600E; ARID1A
168753-222-R-J1-PDC	Bladder	U2AF1 L5V; BRCA1; ATM; FGFR3; KIT
171881-019-R-J1-PDC	Breast	ATXN1, BRCA1, MUTYH, PIK3CA, MLH1, MSH3, ATXN2, TBX3, CDH1, TP53, BRIP1, KEAP1,PARP1 AMP; ATM, PALB2
186277-243-T-J2-PDC	Colon	BRCA2, KRAS G12D, PIK3CA, ATM, ARID1A, ARID4B, DNMT3A, TGFBR2, ATR, NF1
188146-221-R-J1-PDC	Colon	BRCA2, XRCC1, ATM, BRAF V600E; TGFBR2
217524-143-R1-J4-PDC	Pancreas	KRAS G12R; ARID1A; SMAD4; STK11
227483-062-R1-J1-PDC	Pancreas	KRAS G12V; ARID4B; SMAD4; MAP2K4
233499-124-R-J2-PDC	Colon	DYRK1B, KRAS A146T; ATR; MTOR; JAK1; APC
233499-124-R-J3-PDC	Colon	BRCA2, ATR, KRAS, MTOR, JAK1, APC, RAD50, KMT2C, CDKN1B, KMT2D, TP53
242566-281-R-J2-PDC	Pancreas	KRAS G12D, ARID1A
276233-004-R-J1-PDC	Melanoma	APC, KRAS G12S, RB1, ERBB4
282377-053-R-J1-PDC	Colon	POLQ, PARP1, ATR, APC, BRAF V600E; RAD50
292921-168-R-J2-PDC	Pancreatic	KRAS G12D, ARID1B, ARID1A, DNMT3A, APC, MET, FANCC, BRCA2, TP53,
299254-011-R-J1-PDC	Melanoma	CLK2, DYRK3, KRAS G12C, ARID1A, BRAF, APC, ATR, ATM,
317291-083-R-J1-PDC	MPNST	BRCA2, LATS1
323965-272-R-J2-PDC	Pancreas	KRAS G12C, ARID1A, FLT3
324938-238-R-PDC	Bladder	ERBB2, SMAD2, CREBBP, ERBB2, SMAD2, TP53
328373-195-R-J1-PDC	Head and Neck	CLK3, DYRK1B, RB1, RAD50
349418-098-R-PDC	NSCLC	BRAF V600E, ARID1A
377384-186-R-J1-PDC	Pancreas	KRAS G12D, ARID1A, RAD51B
379773-124-R-J2-PDC	Endometrial	ARID1A, FGFR2, PTEN
381356-305-R-J1-PDC	Colon	POLQ, MTOR, APC, TP53
422866-222-R5-J1-PDC	Pancreas	KRAS G12C, EGFR, SMAD3, BRCA1,TP53
435261-313-R-J1-PDC	Colon	APC, PIK3CA, KRAS G12V, RB1
439559-082-T-J2-PDC	Colon	BRCA1, BRCA2 AMP, APC, KRAS G12D
454973-116-R3-J5-PDC	Pancreas	DYRK1B, KRAS G12D, BRCA1, TP53
485176-168-R4-J1-PDC	Pancreas	TERT AMP, BRAF V600E, TGFBR2
485368-065-R4-J2-PDC	Pancreas	DYRK1B, KRAS G12V, ARID1A
496974-208-R-J2-PDC	Pancreas	U2AF1, spliceosome mutations, PTPRT
519858-162-T-J1-PDC	Colon	TERT, POLQ, APC, KRAS G12V, TP53
521955-158-R2-J5-PDC	Pancreatic	KRAS G12D, ATR, SMARCA4, ATR, TP53
556581-035-R-J1-PDC	Ovarian	ARID1B, ARID1A, PIK3CA
565232-114-T-J1-PDC	Bladder	APC, SMAD4, RUNX1
596521-263-R-J1-PDC	MPNST	TERT AMP 2; TP53
598228-144-R-J1-PDC	Endometrial	PIK3CA
598228-144-R-J1-PDC	Endometrial	PIK3CA, TP53
616215-338-R-J1-PDC	Colon	DYRK1A, DYRK1B, KRAS G12S, BRAF V600E, ARID1A, ATR
628569-122-R-J1-PDC	Head and Neck	SF3B1, ATM
633275-114-R-J1-PDC	Endometrial	PALB2 frameshift del, KRAS G12A, BRCA2, KEAP1
636577-100-R-J1-PDC	Endometrial	TERT, POLQ, TP53
648629-189-R-J1-PDC	Bladder	APC, PIK3CA, KLF2
653999-131-R-J2-PDC	NSCLC	BRCA1, BRCA2, KRAS G13D, TP53
695427-040-R-J1-PDC	Colon	BRCA2 AMP, APC, KRAS G12D, TP53
743489-274-T-PDC	Renal Cell	ARID1A, PIK3CA, BRCA1, PTEN, KMT2D, TP53
777334-354-R1-J3-PDC	Pancreas	POLR1A, BRCA2, KRAS G12D, TP53
786-0	Renal Cell	ATM, ERCC3, HIF1A, KMT2D, MTOR, PDGFRA, PDGFRB, PTEN, SMAD2, TOP1, TP53, VHL
817829-284-R-J1-PDC	Colon	DYRK1A, DYRK1B, KRAS G12S, BRAF V600E, ARID1A, ATR
825966-067-R-J1-PDC	Colon	POLR1A, BRCA2, EGFR, BRAF V600E, TFGBR2, PIK3CA
855422-203-R-PDC	Bladder	PIK3CA, DICER1, TP53
857933-349-R-J2-PDC	Colon	BRCA2, PARP1, ATR, ATM, BRAF V600E, APC, TGFBR2
874868-142-R-J2-PDC	Head and Neck	DYRK2, DYRK4, FGFR3, SMAD4
876135-273-R-J2-PDC	Melanoma	CLK2, NRAS, NF1
876862-298-R-J1-PDC	Endometrial	CLK2, SF3B1, ARID1A, PIK3CA
883617-216-R-PDC	Bladder	EGFR, HRAS, KMT2C, ATM, KDM6A
885512-296-R-J2-PDC	Breast	TP53, STK11, KMT2C, KMT2D, RB1
885724-159-R-J1-PDC	Pancreas	BRCA1 AMP, KRAS G12V, TP53, ERBB2, SMAD4
922993-354-T-J2-PDC	Endometrial	ATM, BRCA2, TERT AMP, ATM, BRCA2
947758-054-R-J2-PDC	Colon	CLK3, DYRK1A, ARID1A, ATR, BRAF V600E
966289-007-R4-J1-PDC	Pancreas	BRAF, TP53
996289-038-R-J1-PDC	Colon	CLK4, DYRK3, KRAS G12D, APC
997537-175-T-J1-PDC	Colon	BRCA2, XRCC1, ATM, ATR, BRAF V600E
A375	Melanoma	BRAF V600G, CDKN2A, TERT
A498	Renal Cell	FGFR2, PDGFRA, POU2F2, PIK3CA, VHL
IGROV1	Ovary	BRCA1, BRCA2, PIK3CA, RB1, SMAD4, TP53
K24384-001-R-PDC	Pancreas	KRAS G12V, TP53, NOTCH1, ATM
K-562	Leukemia	BCR-ABL, ASXL1, HOXA9, NOTCH1, TP53
LG0703-F948-PDC	NSCLC	EGFR, TP53
MDA-MB-231	Breast, TNBC	AR, ATM, BRAF, BRCA1, EGFR, KRAS, MYCL, NF1, NF2, NTRK1, PDGFRA, TMPRSS2, TP53
MDA-MB-468	Breast, TNBC	ATR, BRCA2, BRD4, DDR2, FLT3, JAK1, KMT2C, NF1, NTRK3, PIK3CA, PTEN, TOP1, TP53
NCI/ADR-RES	Ovary	ARID1A, BRD4, CTNNB1, ERBB2, KMT2A, KRAS, RUNX1, TP53
NCI-H1876	SCLC (etop sensit)	RB1, ARID1B, ERCC2, ERCC4, KDM6A, KMT2D, MYB, NOTCH2, NRAS, NTRK3, PDGFRA, PDGFRB, TP53
NCI-H196	SCLC (etop resist)	RB1, BCL2, BRCA2, BRD3, BRD4, ERBB4, ERCC5, JAK2, KDM5A, MTOR, POLE, PTEN, SMAD2, TP53, USP6, WRN
NCI-H211	SCLC (etop sensit)	RB1wt, APOBEC3, FANCD2, MTOR, NOTCH2, PDGFRA, PIK3CA, POLQ, POU2AF1, SMARCA4, TP53
OVCAR-5	Ovary	CREBBP, DICER1, ERCC2, EZH2, KRAS, NF1, NTRK3, TGFBR2
SUM149PT	Breast, TNBC	BRAF V600G, CDKN2A, TERT
SW 1271	SCLC (etop resist)	RB1, ABL1, ATM, BRAF, BRCA1, BRCA2, EGFR, ERCC2, FANCA, FGFR1, FGFR2, JAK2, JAK3, KDM5A, KEAP1, MYC, NF1, NRAS, PALB2, PIK3CB, PIK3R1, POU2AF1, TERT, TOP1, TP53, WRN

The disease type and key genetic aberrations for each line are shown. Some lines are from the DCTD PDMR (https://pdmr.cancer.gov/)

**Cell Culture**. All cells were maintained in an incubator at 37 °C and 5% CO_2_ with 95% humidity. The PDC lines were cultured according to standard operating procedures established by the NCI PDMR (https://pdmr.cancer.gov). Briefly, all PDCs were thawed and cultured in Matrigel-coated flasks prepared with a working solution of 1X Ham’s F-12 nutrient mix, without supplementation (Invitrogen, Waltham, MA, cat. 11765054), 100 U/mL penicillin-streptomycin (Invitrogen, cat. 15140122), and 2.5% Matrigel (Corning Inc., Corning, NY, cat. 354248) for the first three passages. All PDCs were cultured in complete DMEM/F-12 media containing advanced DMEM/F-12 (Invitrogen, cat. 12634028), 4.9% defined fetal bovine serum, heat inactivated (HyClone Laboratories Inc., Logan, UT, cat. SH30070.03HI), 389 ng/mL hydrocortisone (Sigma-Aldrich, cat. H4001), 9.7 ng/mL human EGF recombinant protein (Invitrogen, cat. PHG0313), 23.4 µg/mL adenine (Sigma-Aldrich, cat. A2786), 97.3 U/mL penicillin-streptomycin (Invitrogen, cat. 15140122), 1.9 mM L-glutamine (Invitrogen, cat. 25030081), and 9.7 µM Y-27,632 dihydrochloride (Tocris Bioscience, Bristol, United Kingdom, cat. 1254). The PDCs were cultured in complete DMEM/F12 media without 10 µM Y-27,632 dihydrochloride for at least two passages prior to the screen, unless specified otherwise (Table 3). The established cell lines were cultured in RPMI-1640 medium, HEPES (Invitrogen, cat. 22400105) with 10% defined fetal bovine serum (HyClone Laboratories Inc., cat. SH30070.03) and 2 mM L-glutamine (Invitrogen, cat. 25030081), unless specified otherwise (Table 3). The pooled donor HUVEC and hMSC were cultured in endothelial cell growth medium 2 (PromoCell, Heidelberg, Germany, cat. C-22011) and mesenchymal stem cell growth medium 2 (PromoCell, cat. C-28009). For all experiments, HUVEC and hMSCs were used at passages ≤ 5, while the malignant cell lines were used at passages ≤ 15. Samples of the cell lines were collected at regular intervals throughout the screening process for short tandem repeat (STR) profiling and mycoplasma testing by Labcorp (Laboratory Corporation of America Holdings, Burlington, NC, formerly known as Genetica DNA Laboratories) to confirm their authenticity and integrity.

**Table 3:** Growth media and number of tumor cells, endothelial cells and human mesenchymal stem cells plated per well plated to form the mct-spheroids tested spheroids for response to cirtuvivint, CC-671 or Iadademstat alone and in simultaneous combination with the compounds in Table 1

Malignant cell line	Malignant Cell Growth Medium	Malignant Cells per well	HUVEC^a^ per well	hMSC^b^ per well
138582-337-R-J1	Complete DMEM/F12 Media-Y	5000	2083	1250
156681-154-R-J1	Complete DMEM/F12 Media-Y	1250	521	313
168753-222-R-J1	Complete DMEM/F12 Media-Y	5000	2083	1250
171881-019-R-J1	Complete DMEM/F12 Media-Y	2500	1042	625
186277-243-T-J2	Complete DMEM/F12 Media-Y	1250	521	313
188146-221-R-J1	Complete DMEM/F12 Media-Y	2500	1042	625
192522-019-R-J2	Complete DMEM/F12 Media-Y	5000	2083	1250
217524-143-R1-J4	Complete DMEM/F12 Media-Y	1250	521	313
227483-062-R1-J1	Panc + FBS	1250	521	313
233499-124-R-J2	Complete DMEM/F12 Media-Y	1250	521	313
233499-124-R-J3	Complete DMEM/F12 Media-Y	1250	521	313
242566-281-R-J2	Complete DMEM/F12 Media-Y	1250	521	313
276233-004-R-J1	Complete DMEM/F12 Media-Y	2500	1042	625
282377-053-R-J1	Complete DMEM/F12 Media-Y	625	260	156
292921-168-R-J2	Complete DMEM/F12 Media-Y	625	260	156
299254-011-R-J1	Complete DMEM/F12 Media-Y	1250	521	313
317291-083-R-J1	Complete DMEM/F12 Media-Y	313	130	78
323965-272-R-J2	Complete DMEM/F12 Media-Y	1250	521	313
324938-238-R	Complete DMEM/F12 Media-Y	1250	521	313
328373-195-R-J1	Complete DMEM/F12 Media-Y	625	260	156
349418-098-R	Complete DMEM/F12 Media-Y	313	130	78
377384-186-R-J1	Complete DMEM/F12 Media-Y	313	130	78
379773-124-R-J2	Complete DMEM/F12 Media-Y	313	130	78
381356-305-R-J1	Complete DMEM/F12 Media-Y	1250	521	313
422866-222-R5-J1	Complete DMEM/F12 Media-Y	1250	521	313
435261-313-R-J1	Complete DMEM/F12 Media-Y	1250	521	313
439559-082-T-J2	Complete DMEM/F12 Media-Y	2500	1042	625
454973-116-R3-J5	Complete DMEM/F12 Media-Y	1250	521	313
485176-168-R4-J1	Panc + FBS	1250	521	313
485368-065-R4-J2	Complete DMEM/F12 Media-Y	2500	1042	625
496974-208-R-J2	Complete DMEM/F12 Media-Y	2500	1042	625
519858-162-T-J1	Complete DMEM/F12 Media-Y	2500	1042	625
521955-158-R2-J5	Complete DMEM/F12 Media-Y	1250	521	313
556581-035-R-J1	Complete DMEM/F12 Media-Y	2500	1042	625
565232-114-T-J1	Complete DMEM/F12 Media-Y	1250	521	313
596521-263-R-J1	Complete DMEM/F12 Media-Y	1250	521	313
598228-144-R-J1	Complete DMEM/F12 Media-Y	2500	1042	625
616215-338-R-J1	Complete DMEM/F12 Media-Y	625	260	156
628569-122-R-J1	Complete DMEM/F12 Media-Y	2500	1042	625
633275-114-R-J1	Complete DMEM/F12 Media-Y	1250	521	313
636577-100-R-J1	Complete DMEM/F12 Media-Y	2500	1042	625
648629-189-R-J1	Complete DMEM/F12 Media-Y	625	260	156
653999-131-R-J2	Complete DMEM/F12 Media-Y	1250	521	313
695427-040-R-J1	Complete DMEM/F12 Media-Y	2500	1042	625
743489-274-T	Complete DMEM/F12 Media-Y	2500	1042	625
777334-354-R1-J3	Complete DMEM/F12 Media-Y	2500	1042	625
817829-284-R-J1	Complete DMEM/F12 Media-Y	625	260	156
825966-067-R-J1	Complete DMEM/F12 Media-Y	1250	521	313
855422-203-R-J1	Complete DMEM/F12 Media-Y	313	130	78
857933-349-R-J2	6 C/COLON 1B -Y^c^	2500	1042	625
874868-142-R-J2	Complete DMEM/F12 Media-Y	2500	1042	625
876135-273-R-J2	Complete DMEM/F12 Media-Y	1250	521	313
876862-298-R-J1	6E + FBS + Y	2500	1042	625
883617-216-R-J1	Complete DMEM/F12 Media-Y	625	260	156
885512-296-R-J2	Complete DMEM/F12 Media-Y	313	130	78
885724-159-R-J1	Complete DMEM/F12 Media-Y	2500	1042	625
922993-354-T-J3	Complete DMEM/F12 Media-Y	2500	1042	625
947758-054-R-J2	6B/Colon 1 A + FBS + Y	625	260	156
966289-007-R4-J1	Complete DMEM/F12 Media-Y	313	130	78
996289-038-R-J1	Complete DMEM/F12 Media-Y	2500	1042	625
997537-175-T-J1	Complete DMEM/F12 Media-Y	2500	1042	625
786-0	RPMI-1640 + 10% FBS	313	130	78
A375	HITES DMEM/F12 + 5% FBS	313	130	78
A498	RPMI-1640 + 10% FBS	1250	521	313
IGROV1	RPMI-1640/10% FBS/1% L-Glut	313	130	78
K24384-001-R-PDC	Complete DMEM/F12 Media-Y	625	260	156
K562	RPMI-1640/10% FBS/1% L-Glut	313	130	78
LG0703-F948-PDC	Complete DMEM/F12 Media-Y	625	260	156
MDA-MB-231	RPMI-1640/10% FBS/1% L-Glut	625	260	156
MDA-MB-468	RPMI-1640/10% FBS/1% L-Glut	625	260	156
NCI/ADR-RES	RPMI-1640 + 10% FBS	313	130	78
NCI-H1876	HITES DMEM/F12 + 5% FBS	1250	521	313
NCI-H196	RPMI-1640 + 10% FBS	2500	1042	625
NCI-H211	RPMI-1640/10% FBS/1% L-Glut	313	130	78
OVCAR-5	RPMI-1640 + 10% FBS	1250	521	313
SUM149PT	HAM’s F12 + 5% FBS	1250	521	313
SW 1271	HITES DMEM/F12 + 5% FBS	625	260	156

The disease type and selected genetic properties for each line are shown. Cell lines are from the DCTD PDMR (https://pdmr.cancer.gov/)

^a^Human umbilical vein endothelial cells (HUVEC)

^b^Human mesenchymal stem cells (hMSC)

^c^Detailed descriptions of 6 C/COLON 1B -Y and Breast #2 -Y are available at (https://pdmr.cancer.gov/)

**High-throughput Drug Combination Screening.** Prior to their inoculation into microplates, malignant cells, HUVEC, and hMSC were removed from T flasks using TrypLE express (Invitrogen, cat. 12605036) and harvested by centrifugation for 5 min at 233 × g. Following removal of the supernatant, the cells were resuspended in fresh medium and counted using a Cellometer auto T4 bright field cell counter (Nexcelom, Lawrence, MA) and trypan blue to distinguish viable cells. Multi-cell type (mct) tumor spheroids were grown from the mixture of three cell types: 60% malignant cells, 25% HUVECs, and 15% hMSCs as described previously shown in Supplemental Fig. 1 [2]. Mixed cell suspensions of 50 µL were dispensed into the wells of 384-well black/clear round-bottom ULA spheroid microplates (Corning Inc., cat. 3830). Following inoculation, the microplates were transferred to an incubator (Thermo Fisher Scientific, Waltham, MA) and maintained at 37 °C and 5% CO_2_ with 95% humidity. Three days after inoculation, test agents or controls were delivered to the wells of microplates. The approved and investigational anticancer agents, prepared as 800× stock solutions, were subsequently transferred in 62.5 nL volumes to the appropriate wells of microplates using an I.DOT non-contact dispenser (DISPENDIX, Stuttgart, Germany) to achieve a 1x final concentration. All anticancer agents and their combinations were tested in quadruplicate. Additionally, each microplate included a DMSO vehicle control (*n* = 16) and a cytotoxicity control (1 µM staurosporine and 3 µM gemcitabine, *n* = 20). After delivery of the test agents and controls, the microplates were returned to the incubator for 7 days. Ten days after inoculation, the assay was completed with the addition of 20 µL CellTiter-Glo 3D (Promega, Madison, WI, cat. G9683) to each well. Next, the microplates were placed on a microplate shaker for 5 min. After 25 min of incubation at room temperature, luminescence was measured as a surrogate indicator of cell viability using a PHERAstar FSX microplate reader (BMG LABTECH, Cary, NC) [21, 22].

**Fig. 1:**
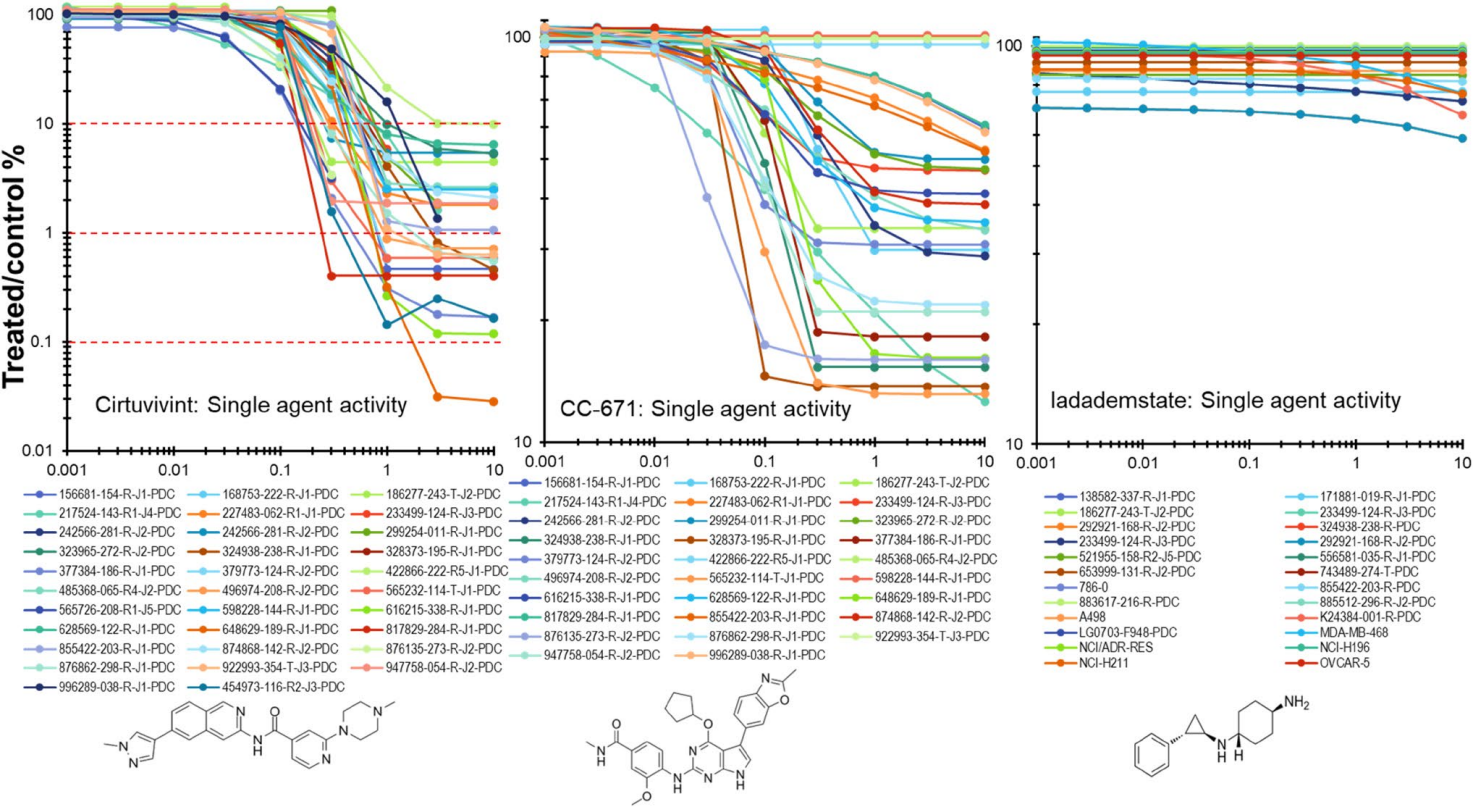
Single agent concentration response data for the pan-CLK inhibitor cirtuvivint, the CLK/TTK inhibitor CC-671and the KDM1A inhibitor idademstat in 26 human tumor cell lines over a 9-point concentration range covering 4-logs of concentration after a 7-day exposure time. The chemical structures of cirtuvivint, CC-671, and iadademstat are shown

**Data analysis**. Luminescence measurements from the screen were exported as comma separated values (CSV) files and imported into custom Excel spreadsheets (Microsoft, Redmond, WA) for analysis. The raw luminescence data were evaluated for quality control, filtered for outliers, and converted to percent viability by normalizing to the DMSO (vehicle-treated) control. Concentration-response data were fit to the four-parameter logistic equation using the Solver Add-In in Excel. The Bliss independence model states that if two drugs have independent activities, then the viability for the combination is equal to the product of the viability of the two single agents [22, 23]. Synergy between two compounds was indicated by a lower observed percent viability than predicted by the Bliss independence model, whereas antagonism was indicated by a greater observed percent viability than predicted. The mean and statistical significance of Bliss independence scores for each drug combination-model across all concentrations and biological replicates were evaluated [23]. Response surface maps were generated using the MATLAB web application where blue indicates synergy and red indicates antagonism.

**Data availability**. All data are accessible via the PubChem BioAssay public database (AID 1918931; AID1918932; AID1918933; AID1918930; AID1918934; AID1918935; AID1918936; AID1918937; AID1918938; AID8939; AID1918942; AID8944; AID1918943; AID1918945; AID1918946; AID1918947; AID1918949; AID1918948; AID8950; AID1918951; AID1918952; AID1918953; AID1918954; AID1918955; AID1918957; AID1918958; AID1918940; AID1918956; AID8941; AID2060627; AID 2060626; AID 2060625; AID 2060624; AID 2060623; AID 2060613; AID 2060622; AID 2060619; AID 2060621; AID 2060620; AID 2060616; AID 2060604; AID 2060603; AID 2060618; AID 2060617; AID 2060615; AID 2060612; AID 2060614; AID 2060610; AID 2060611; AID 2060602; AID 2060599; AID 2060609; AID 2060601; AID 2060608; AID 2060607; AID 2060606; AID 2060605; AID 2060600 (Table 4).

## Results

Single agent concentration response data for two investigational CLK inhibitors cirtuvivint and CC-671, and an investigational LSD1 inhibitor iadademstat, are shown in Fig. 1. The compounds were tested 26 human tumor cells lines grown as mct-spheroids in 9-point concentration response at concentrations spanning 4-logs with an exposure time of 7 days. Cirtuvivint was the most potent cytotoxin with IC_90_ concentrations between 0.2 and 10 µM. The LSD-1 inhibitor iadademstat was assessed in mct-spheroids in 29 cell lines including PDMR lines and established lines. The most responsive lines were the NCI-H211 SCLC, two triple-negative breast cancer lines MDA-MB-231 and MDA-MB-468, a pancreatic carcinoma line 292921-168-R-J2, and an ovarian carcinoma line 556581-035-R-J1; however, only the NCI-H211 mct-spheroids reached an IC_50_ at 7.5 µM. Iadademstat was the least cytotoxic and reached an IC_50_ concentration only in the MDA-MB-468 cell line at 10 µM, the highest concentration tested. Cirtuvivint, CC-671 and iadademstat were tested in combination with anticancer drugs and investigational agents in complex mct-spheroids composed of human tumor cells with known genetic alterations, HUVEC and hMSC cells. The drugs and investigational agents tested are listed in Table 1. The genetic variants for key genes in the tumor cell lines are shown on Table 2. Drugs and investigational agents were tested at concentrations beginning at the clinical Cmax concentration or at 10 µM, if the clinical Cmax was not established, decreasing in half-log increments for 6 concentrations.

**Fig. 2:**
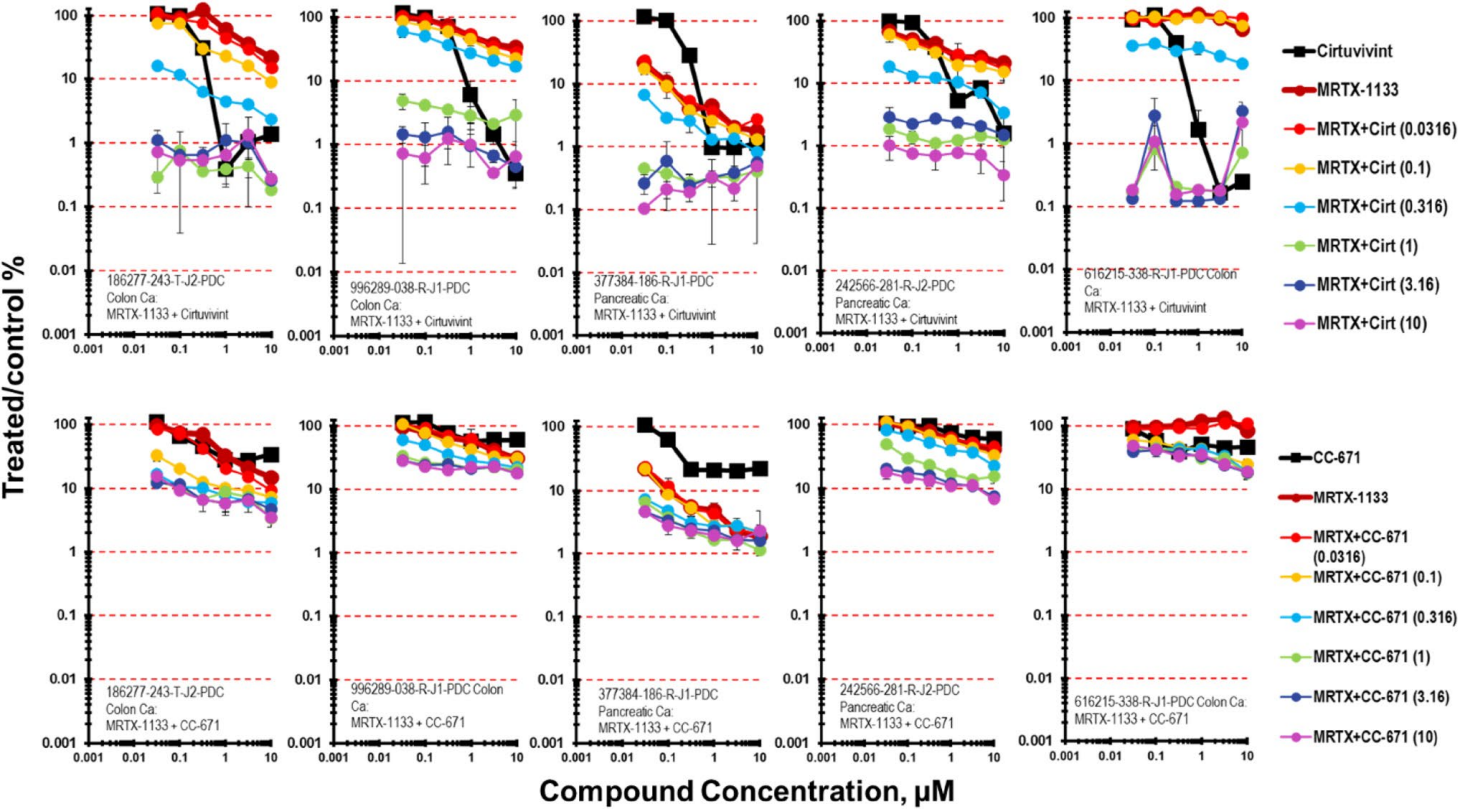
Concentration response data for the pan-CLK inhibitor cirtuvivint or the CLK/TTK inhibitor CC-671 tested in combination with the KRAS G12D selective inhibitor MRTX-1133. Data are shown for five PDMR human tumor cell lines grown as mct-spheroids, 186277-243-T-J2 TP53 wt, KRAS G12D colon carcinoma, 996289-038-R-J1 TP53 mutant, KRAS G12D, CLK4 mutant, DYRK3 mutant colon carcinoma, 377384-186-R-J1 TP53 mutant, KRAS G12D pancreatic carcinoma, 242566-281-R-J2 TP53 mutant, KRAS G12D pancreatic carcinoma and 616215-338-R-J1 TP53 wt, KRAS G12S, BRAF V600E. All data show the mean ± SD (*n* = 4)

The KRAS G12D selective inhibitor MRTX-1133 was assessed in combination with cirtuvivint in four PDMR lines harboring the KRAS G12D mutation and the 616215-338-R-J1 colon carcinoma which harbors a KRAS G12S mutation (Fig. 2) [24]. The PDMR lines with KRAS G12D were among the most responsive to MRTX-1133 as a single agent with 377384-186-R-J1 pancreatic carcinoma mct-spheroids being most responsive (Supplemental Fig. 2). Simultaneous combination of cirtuvivint and MRTX-1133 produced primarily additive cytotoxicity with a few regions of great-than-additive cytotoxicity in the 186277-243-T-J2 colon carcinoma, 996289-038-R-J1 colon carcinoma, 377384-186-R-J1 pancreatic carcinoma and 242566-281-R-J2 pancreatic carcinoma mct-spheroids with up to 2.5-3-logs of cell killing. The 616215-338-R-J1 colon carcinoma harboring a KRAS G12S mutation was also responsive to the combination of cirtuvivint and MTRX-1133. The response of the same five tumor mct-spheroids was assessed after 7-days exposure to CC-671 with MRTX-1133 (Fig. 2). Tumor cell killing was greatest in the 377384-186-R-J1 pancreatic carcinoma complex mct-spheroids reaching 2-logs which was additive by the Bliss independence calculation. Greater-than-additive cell killing was evident under the same conditions for the 377384-186-R-J1 pancreatic carcinoma, the186277-243-T-J2 colon carcinoma, 996289-038-R-J1 colon carcinoma mct-spheroids. The maximal tumor cell killing achieved with CC-671 and MRTX-1133 in the 377384-186-R-J1 pancreatic carcinoma, the 186277-243-T-J2 colon carcinoma, 996289-038-R-J1 colon carcinoma and 616215-338-R-J1 colon carcinoma mct-spheroids was 1-log.

**Fig. 3:**
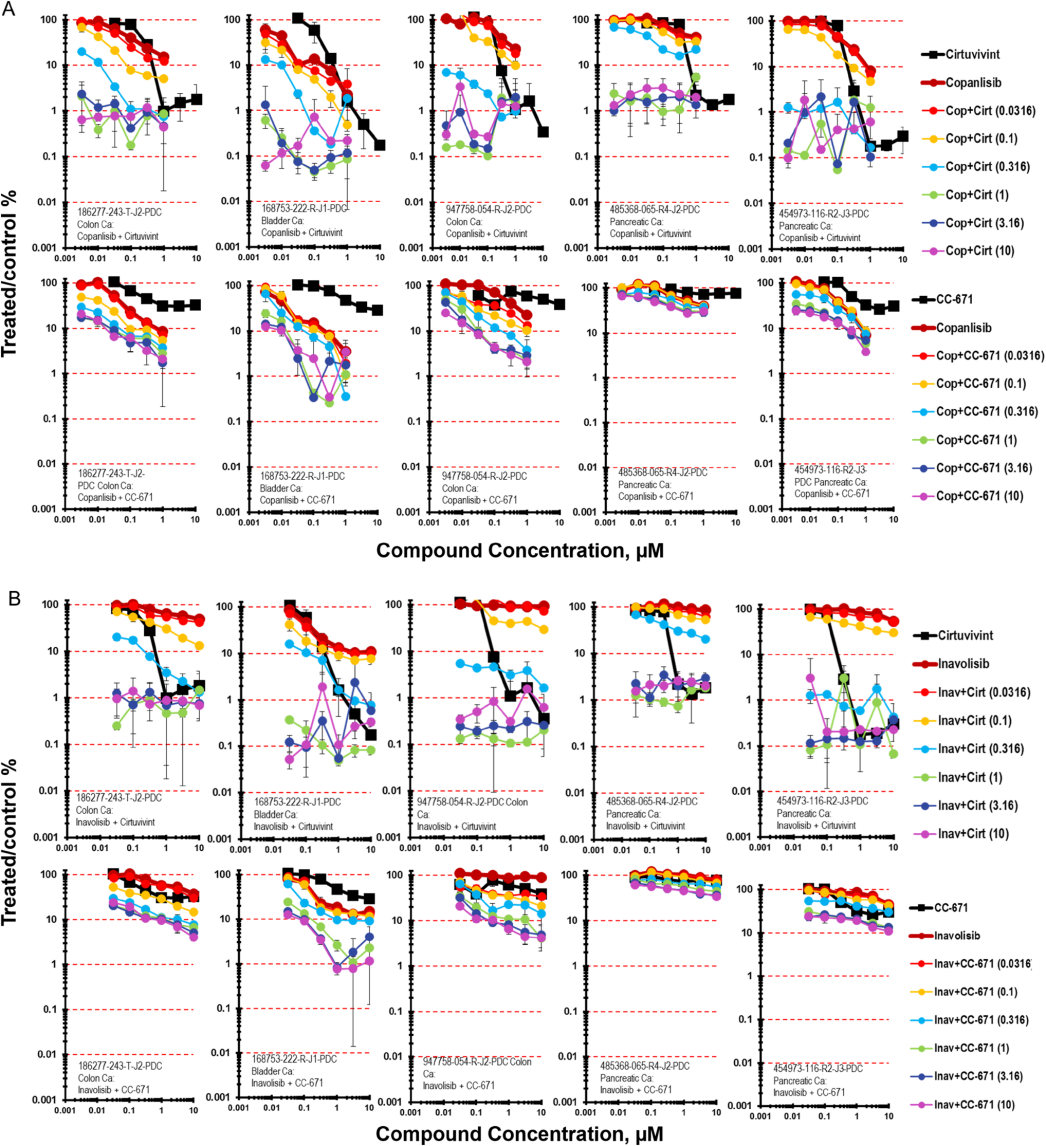
**A** Concentration response data for the pan-CLK inhibitor cirtuvivint or the CLK/TTK inhibitor CC-671 tested in combination with the pan-PI3K inhibitor copanlisib. Data are shown for five PDMR human tumor cell lines grown as complex spheroids, 186277-243-T-J2 colon carcinoma, 168753-222-R-J1 bladder carcinoma, 947758-054-R-J2 colon carcinoma, 485368-065-R4-J2 pancreatic carcinoma and 454973-116-R3-J5 pancreatic carcinoma. All data show the mean ± SD (*n* = 4). **B** Concentration response data for the pan-CLK inhibitor cirtuvivint or the CLK/TTK inhibitor CC-671 tested in combination with the PI3Kalpha selective inhibitor inavolisib. Data are shown for five PDMR human tumor cell lines grown as mct-spheroids, 186277-243-T-J2 colon carcinoma, 168753-222-R-J1 bladder carcinoma, 947758-054-R-J2 colon carcinoma, 485368-065-R4-J2 pancreatic carcinoma and 454973-116-R3-J5 pancreatic carcinoma. All data show the mean ± SD (*n* = 4)

Moving down-stream of RAS, the simultaneous combination of cirtuvivint and the pan-PI3K inhibitor copanlisib was examined in five PDMR cell lines, the 186277-243-T-J2 colon carcinoma, the 168753-222-R-J1 bladder carcinoma, 947758-054-R-J2 colon carcinoma, 485368-065-R4-J2 pancreatic carcinoma and 454973-116-R3-J5 pancreatic carcinoma mct-spheroids with a 7-day exposure (Fig. 3A) [25]. The 168753-222-R-J1 bladder carcinoma mct-spheroids were more responsive to copanlisib as a single agent than were the 186277-243-T-J2 colon carcinoma mct-spheroids. The tumor cell killing was primarily additive in both tumor lines reaching 2-logs for the 186277-243-T-J2 colon carcinoma mct-spheroids and 3-logs for the168753-222-R-J1 bladder carcinoma mct-spheroids. The 947758-054-R-J2 colon carcinoma, 485368-065-R4-J2 pancreatic carcinoma and 454973-116-R3-J5 pancreatic carcinoma mct-spheroids with a 7-day exposure were similarly responsive to the combination of copanlisib and cirtuvivint with cell killing reaching 2- to −3-logs. The same five PDMR cell lines were exposed to CC-671 in simultaneous combination with copanlisib (Fig. 3A). The combination of CC-671 with copanlisib was less cytotoxic than the combination of cirtuvivint with copanlisib in each of the five cell lines tested as mct-spheroids. The combination produced maximal cytotoxicity in the 168753-222-R-J1 bladder carcinoma mct-spheroids with additive to greater-than additive cell killing reaching 2-logs while the same combination produced less than 1-log of cell kiliing in the 485368-065-R4-J2 pancreatic carcinoma mct-spheroids.

The simultaneous combination of cituvivint and the PI3Ka selective inhibitor inavolisib in 186277-243-T-J2 colon carcinoma mct-spheroids resulted in additive to greater-than-additive cytotoxicity reaching a maximum to 2-logs (Fig. 3B) [26]. As determined by the Bliss independence calculation the combination of cirtuvivint and inavolisib produced additive cell killing in the 168753-222-RJ1 bladder carcinoma mct-spheroids reaching 3-logs at the 2 highest concentrations of cirtuvivint across the concentration range of inavolisib. Greater-than-additive cell killing was seen with inavolisib in simultaneous combination with CC-671 in mct-spheroids grown from 947758-054-R-J2 colon carcinoma cells and 186277-243-T-J2 colon carcinoma cells (Fig. 3B). Neither 947758-054-RJ2 colon carcinoma mct-spheroids nor 186277-243-TJ2 colon carcinoma mct-spheroids was very responsive to CC-671 reaching 62% and 70% cell killing, respectively. The combination of CC-671 and inavolisib reached nearly 2-logs of cell killing in both lines with more evidence of greater-than-additive cell killing occurring in the 947758-054-R-J2 colon carcinoma mct-spheroids. Inavolisib was not effective in the 947758-054-R-J2 colon carcinoma mct-spheroids; however, greater-than-additive killing occurred over the 6 × 6 concentration matrix, the same was true of the combination of CC-671 and inavolisib in the 186277-243-TJ2 colon carcinoma mct-spheroids albeit the magnitude was less. Overall, the combination of CC-671 and inavolisib reached 1.5-logs of cell killing in both tumor lines.

**Fig. 4:**
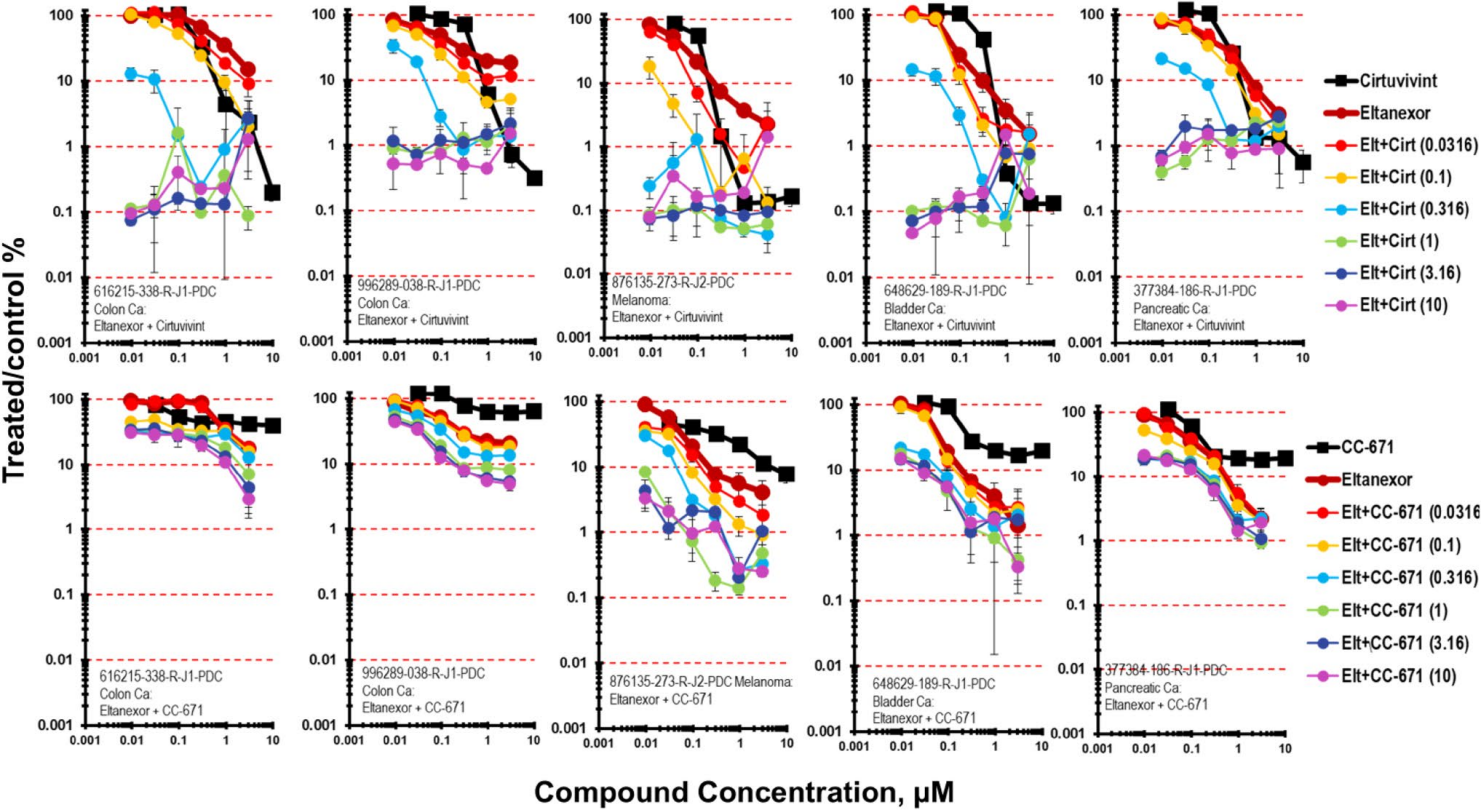
Concentration response data for the pan-CLK inhibitor cirtuvivint or the CLK/TTK inhibitor CC-671 tested in combination with the XPO-1 inhibitor eltanexor. Data are shown for five PDMR human tumor cell lines grown as complex spheroids, 616215-338-R-J1 colon carcinoma, 996289-038-R-J1 colon carcinoma, 876135-273-R-J2 melanoma, 648629-189-R-J1 bladder carcinoma and 377384-186-R-J1-PDC pancreatic carcinoma. All data show the mean ± SD (*n* = 4)

As a single agent the XPO1 inhibitor eltanexor, resulted in 1- to 2-logs of cytotoxicity in mct-spheroids after a 7-day exposure [27, 28]. Five cell lines were selected to highlight the results from the combination of cirtuvivint and eltanexor (Fig. 4). The simultaneous combination of cirtuvivint with eltanexor produced additive to greater-than-additive killing of 616215-338-R-J1 colon carcinoma mct-spheroids. While greater-than-additive cytotoxicity of the combination was observed at moderate concentrations of eltanexor and lower concentrations of cirtuvivint, cell killing of 2- to 3-logs was observed at the higher concentrations of both agents. The simultaneous combination of cirtuvivint and eltanexor produced greater-than-additive cytotoxicity at moderate concentrations of both agents in the 996289-038-R-J1 colon carcinoma mct-spheroids reaching 2.5-logs. The simultaneous combination of cirtuvivint and eltanexor was additive in the 876135-273-R-J2 melanoma, the 648629-189-R-J1 bladder carcinoma and 377384-186-R-J1 pancreatic carcinoma. Additive to less-than additive tumor cell killing occurred over a wide concentration range of both CC-671 and eltanexor in 616215-338-R-J1 colon carcinoma and the 996289-038-R-J1 colon carcinoma mct-spheroids reaching a maximum of about 1.5-logs. The greatest depth of tumor cell killing was observed in the 876135-273-R-J2 melanoma mct-spheroids reaching 3-logs at the two highest concentrations of CC-671 and eltanexor (Fig. 4).

**Fig. 5:**
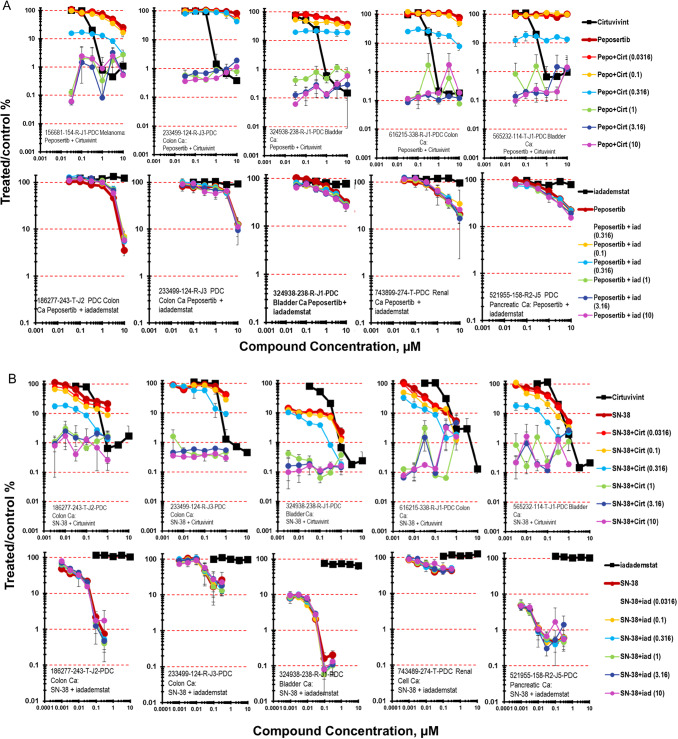
**A** Concentration response data for the pan-CLK inhibitor cirtuvivint or the KDM1A/LSD1 inhibitor idademstat tested in combination with the DNA-PK inhibitor peposertib. Data are shown for five PDMR human tumor cell lines grown as mct-spheroids, 156681-154-R-J1 melanoma or 186277-243-T-J2 colon carcinoma, 233499-124-R-J2 colon carcinoma, 324938-238-R bladder carcinoma, 616215-338-R-J1 colon carcinoma, and 565232-114-T-J1 bladder carcinoma. All data show the mean ± SD (*n* = 4). **B** Concentration response data for the pan-CLK inhibitor cirtuvivint or the KDM1A inhibitor idademstat tested in combination with the topoisomerase 1 inhibitor SN-38. Data are shown for five PDMR human tumor cell lines grown as complex spheroids, 186277-243-T-J2 colon carcinoma, 233499-124-R-J2 colon carcinoma, 324938-238-R bladder carcinoma, 616215-338-R-J1 colon carcinoma, and 565232-114-T-J1 bladder carcinoma. All data show the mean ± SD (*n* = 4)

The DNA-PK inhibitor peposertib was studied in combination with the CLK inhibitor cirtuvivint or with the LSD-1 inhibitor iadademstat (Fig. 5A) [29–31]. While peposertib as a single agent was not highly effective in the five PDMR cell lines highlighted, 1566681-154-R-J1 melanoma, 233499-124-R-J3 and 616215-338-R-J1 colon carcinomas, 324938-238-R-1 and 565232-114-T-J1 bladder carcinomas, the combination of peposertib and cirtuvivint resulted in 2- to 3-logs of cell killing in each of the 5 lines grown as mct-spheroids. In contrast, the combination of peposertib with the LSD-1 inhibitor iadademstat resulted in sub-additive to additive cell killing in the five PDMR lines highlighted, 186277-243-T-J2 and 233499-124-R-J3 colon carcinomas, 324938-238-R-1 bladder carcinoma, 743899-274-T renal cell carcinoma and 521955-158-R2-J5 pancreatic carcinoma (Fig. 5A).

SN-38 is the active metabolite of the prodrug irinotecan, that acts, like irinotecan, by inhibiting topoisomerase I, an enzyme induces a single strand break in DNA to relax the DNA during replication [32, 33]. SN-38 was tested at concentrations up to 1µM during the 7-day exposure period (Fig. 5B). At the lower concentrations of cirtuvivint and moderate to high concentrations of SN-38 marked greater-than-additive tumor cell killing with Bliss additivity scores up to 66 for the 616215-338-R-J1 colon carcinoma, 58 for the 233499-124-R-J3 colon carcinoma, 40 for the 565232-114-T-J1 bladder carcinoma, and 21 for the 186277-243-T-J2 colon carcinoma while, the combination of cirtuvivint and SN-38 produced additive cytotoxicity due to the sensitivity of the 186277-243-T-J2 mct-spheroid to SN-38 which killed 1-log of cells as a single agent (Fig. 5B). By comparison, the combination of iadademstat and SN-38 produced less-than-additive to additive cell killing over the iadademstat and SN-38 concentrations tested.

**Fig. 6:**
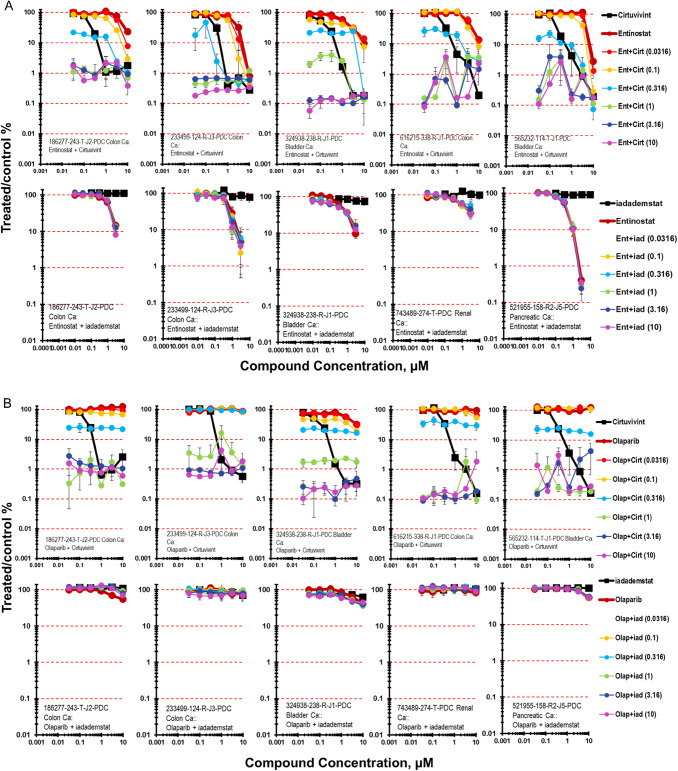
**A** Concentration response data for the pan-CLK inhibitor cirtuvivint or the KDM1A inhibitor idademstat tested in combination with the class I histone deacetylase (HDAC) inhibitor entinostat. Data are shown for five PDMR human tumor cell lines grown as mct-spheroids, 186277-243-T-J2 colon carcinoma, 233499-124-R-J2 colon carcinoma, 324938-238-R bladder carcinoma, 616215-338-R-J1 colon carcinoma, and 565232-114-T-J1 bladder carcinoma. All data show the mean ± SD (*n* = 4). **B** Concentration response data for the pan-CLK inhibitor cirtuvivint or the KDM1A inhibitor idademstat tested in combination with the poly(ADP-ribose) polymerase (PARP) inhibitor olaparib. Data are shown for five PDMR human tumor cell lines grown as complex spheroids, 186277-243-T-J2 colon carcinoma, 233499-124-R-J2 colon carcinoma, 324938-238-R bladder carcinoma, 616215-338-R-J1 colon carcinoma, and 565232-114-T-J1 bladder carcinoma. All data show the mean ± SD (*n* = 4)

The histone deacetylase inhibitor entinostat was tested in combination with cirtuvivint and iadademstat and the results from five PDMR human tumor cell lines grown as mct-spheroids are shown in Fig. 6A [34, 35]. The combination of entinostat with cirtuvivint were additive in the five cell lines highlighted over the concentration ranges of the drugs tested with a 7-day exposure time. A similar result was obtained with the combination of iadademstat with entinostat with the five PDMR cells lines grown as mct-spheroids producing less-than-additive to additive cytotoxicity.

Combinations of cirtuvivint and iadadematat with the PARP1 inhibitor olaparib in five of the PDMR tumor cell lines grown as mct-spheroids resulted in the combination producing sub-additive to additive cytotoxic effects in the 324938-238-R-1 and 565232-114-T-J1 bladder carcinomas and additive to greater-than additive cytotoxicity in the 186277-243-T-J2 and 616215-338-R-J1 colon carcinomas (Fig. 6B) [36]. The combination of iadademstat with olaparib resulted in little cytotoxicity with combinations just reaching an IC_50_ with the highest concentration of both drugs.

**Fig. 7:**
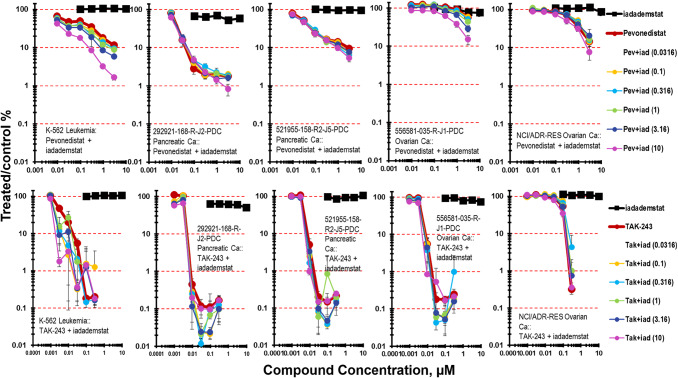
Concentration response data for the KDM1A inhibitor idademstat tested in combination with the NEDD8-activating enzyme (NAE) inhibitor pevonedistat or the ubiquitin activating enzyme (UAE) inhibitor TAK-243. Data are shown for the human K-562 leukemia which does not form spheroids, and four human solid tumor cell lines grown as mct-spheroids, 292921-168-R-J2 pancreatic carcinoma, 521955-158-R2-J5 pancreatic carcinoma, 556581-035-R-J1 ovarian carcinoma, and NCI/ADR-RES ovarian carcinoma. All data show the mean ± SD (*n* = 4)

Iadademstat was tested with the NEDD8-activating enzyme (NAE) inhibitor pevonedistat (Fig. 7). Pevonedistat prevents the activation of cullin-RING E3 ligases thereby blocking the ubiquitination and proteasomal degradation of cellular proteins causing a build-up of proteins leading to cell death [37–41]. The K-562 leukemia was responsive to pevonedistat reaching 1 log of cytotoxicity at the highest concentration (3 µM) tested. There was an iadademstat concentration dependent increase in cytotoxicity with iadademstat and pevonedistat reaching 2-logs at 3 µM pevonedistat (Fig. 7). Of the two PDMR pancreatic carcinoma lines, the less responsive the 521955-158-R2-J5 pancreatic carcinoma mct-spheroids reached > 1-log, while the 292921-186-R-J2 pancreatic carcinoma mct-spheroids reached 2-logs of cell killing at higher pevonedistat concentrations. The OVCAR-5, and NCI/ADR-RES ovarian carcinoma mct- spheroids were responsive to pevonedistat reaching 1-log at 3µM pevonedistat. Combination of pevonedistat with iadademstat did not increase OVCAR-5 or NCI/ADR-RES mct-spheroid killing compared with pevonedistat alone.

TAK-243 is an inhibitor of the (UAE). UAE is the primary E1 enzyme regulating the ubiquitin conjugation cascade [42, 43]. The binding of TAK-243 to UAE prevents protein ubiquitination resulting in protein accumulation (proteotoxic stress) and leading to cell death. The K-562 leukemia was responsive to TAK-243 with 3-logs of cytotoxicity at the highest concentration of TAK-243 (0.3 µM) tested (Fig. 7). Iadademstat with TAK-243 resulted in an iadademstat concentration dependent increase in K-562 leukemia cell cytotoxicity at the lower concentrations of TAK-243 compared with TAK-243 alone. Two of the PDMR pancreatic carcinoma, 292921-186-R-J2 and 521955-158-R2-J5, were as responsive to TAK-243 as was the K-562 leukemia reaching 3-logs of cytotoxicity. Iadademstat with TAK-243 increase in cytotoxicity in the two pancreatic carcinoma mct-spheroids compared with TAK-243. TAK-243 and iadademstat produced a modest increase in cytotoxicity. The PDMR 556581-035-R-J1 ovarian carcinoma was highly responsive to TAK-243 reaching 3-logs of cytotoxicity. Iadademstat with TAK-243 increased cytotoxicity compared with TAK-243 alone at the mid-range concentrations. The NCI/ADR-RES ovarian carcinoma mct-spheroids were responsive to TAK-243 reaching 2-logs of cytotoxicity. Iadademstat with TAK-243 was less cytotoxic than TAK-243 as a single agent in the NCI/ADR-RES cell line. The OVCAR-5 cell line was responsive to TAK-243 reaching nearly 2-logs of cell killing. The combination of iadademstat and TAK-243 was modestly more cytotoxic than TAK-243.

**Table 4:** PubChem data files for the LSD1 screen and the CLK screen

File Name	Group	REGID	AID	Link to access data
Anticancer human tumor 138582-337-R-J1-PDC cell line growth inhibition	Combinations_Synergy_Screen_LSD1	CSSLSD1_01	1,918,931	https://pubchem.ncbi.nlm.nih.gov/bioassay/1918931
Anticancer human tumor 171881-019-R-J1-PDC cell line growth inhibition	Combinations_Synergy_Screen_LSD1	CSSLSD1_02	1,918,932	https://pubchem.ncbi.nlm.nih.gov/bioassay/1918932
Anticancer human tumor 186277-243-T-J2-PDC cell line growth inhibition	Combinations_Synergy_Screen_LSD1	CSSLSD1_03	1,918,933	https://pubchem.ncbi.nlm.nih.gov/bioassay/1918933
Anticancer human tumor 233499-124-R-J3-PDC cell line growth inhibition	Combinations_Synergy_Screen_LSD1	CSSLSD1_04	1,918,930	https://pubchem.ncbi.nlm.nih.gov/bioassay/1918930
Anticancer human tumor 292921-168-R-J2-PDC cell line growth inhibition	Combinations_Synergy_Screen_LSD1	CSSLSD1_05	1,918,934	https://pubchem.ncbi.nlm.nih.gov/bioassay/1918934
Anticancer human tumor 324938-238-R-J1-PDC cell line growth inhibition	Combinations_Synergy_Screen_LSD1	CSSLSD1_06	1,918,935	https://pubchem.ncbi.nlm.nih.gov/bioassay/1918935
Anticancer human tumor 349418-098-R-PDC cell line growth inhibition	Combinations_Synergy_Screen_LSD1	CSSLSD1_07	1,918,936	https://pubchem.ncbi.nlm.nih.gov/bioassay/1918936
Anticancer human tumor 521955-158-R2-J5-PDC cell line growth inhibition	Combinations_Synergy_Screen_LSD1	CSSLSD1_08	1,918,937	https://pubchem.ncbi.nlm.nih.gov/bioassay/1918937
Anticancer human tumor 556581-035-R-J1-PDC cell line growth inhibition	Combinations_Synergy_Screen_LSD1	CSSLSD1_09	1,918,938	https://pubchem.ncbi.nlm.nih.gov/bioassay/1918938
Anticancer human tumor 653999-131-R-J2-PDC cell line growth inhibition	Combinations_Synergy_Screen_LSD1	CSSLSD1_10	1,918,939	https://pubchem.ncbi.nlm.nih.gov/bioassay/1918939
Anticancer human tumor 743489-274-T-PDC cell line growth inhibition	Combinations_Synergy_Screen_LSD1	CSSLSD1_11	1,918,942	https://pubchem.ncbi.nlm.nih.gov/bioassay/1918942
Anticancer human tumor 786-0 cell line growth inhibition	Combinations_Synergy_Screen_LSD1	CSSLSD1_12	1,918,944	https://pubchem.ncbi.nlm.nih.gov/bioassay/1918944
Anticancer human tumor 855422-203-R-J1-PDC cell line growth inhibition	Combinations_Synergy_Screen_LSD1	CSSLSD1_13	1,918,943	https://pubchem.ncbi.nlm.nih.gov/bioassay/1918943
Anticancer human tumor 883617-216-R-J1-PDC cell line growth inhibition	Combinations_Synergy_Screen_LSD1	CSSLSD1_14	1,918,945	https://pubchem.ncbi.nlm.nih.gov/bioassay/1918945
Anticancer human tumor 885512-296-R-J2-PDC cell line growth inhibition	Combinations_Synergy_Screen_LSD1	CSSLSD1_15	1,918,946	https://pubchem.ncbi.nlm.nih.gov/bioassay/1918946
Anticancer human tumor A498 cell line growth inhibition	Combinations_Synergy_Screen_LSD1	CSSLSD1_16	1,918,947	https://pubchem.ncbi.nlm.nih.gov/bioassay/1918947
Anticancer human tumor K24384-001-R-PDC cell line growth inhibition	Combinations_Synergy_Screen_LSD1	CSSLSD1_17	1,918,949	https://pubchem.ncbi.nlm.nih.gov/bioassay/1918949
Anticancer human tumor K-562 cell line growth inhibition	Combinations_Synergy_Screen_LSD1	CSSLSD1_18	1,918,948	https://pubchem.ncbi.nlm.nih.gov/bioassay/1918948
Anticancer human tumor K57222-313-R-J1-PDC cell line growth inhibition	Combinations_Synergy_Screen_LSD1	CSSLSD1_19	1,918,950	https://pubchem.ncbi.nlm.nih.gov/bioassay/1918950
Anticancer human tumor K60290-347-R-J1-PDC cell line growth inhibition	Combinations_Synergy_Screen_LSD1	CSSLSD1_20	1,918,951	https://pubchem.ncbi.nlm.nih.gov/bioassay/1918951
Anticancer human tumor LG0703-F948-PDC cell line growth inhibition	Combinations_Synergy_Screen_LSD1	CSSLSD1_21	1,918,952	https://pubchem.ncbi.nlm.nih.gov/bioassay/1918952
Anticancer human tumor MDA-MB-231/ATCC cell line growth inhibition	Combinations_Synergy_Screen_LSD1	CSSLSD1_22	1,918,953	https://pubchem.ncbi.nlm.nih.gov/bioassay/1918953
Anticancer human tumor MDA-MB-468 cell line growth inhibition	Combinations_Synergy_Screen_LSD1	CSSLSD1_23	1,918,954	https://pubchem.ncbi.nlm.nih.gov/bioassay/1918954
Anticancer human tumor NCI/ADR-RES cell line growth inhibition	Combinations_Synergy_Screen_LSD1	CSSLSD1_24	1,918,955	https://pubchem.ncbi.nlm.nih.gov/bioassay/1918955
Anticancer human tumor NCI-H1876 cell line growth inhibition [LSD1]	Combinations_Synergy_Screen_LSD1	CSSLSD1_25	1,918,957	https://pubchem.ncbi.nlm.nih.gov/bioassay/1918957
Anticancer human tumor NCI-H196 cell line growth inhibition [LSD1]	Combinations_Synergy_Screen_LSD1	CSSLSD1_26	1,918,958	https://pubchem.ncbi.nlm.nih.gov/bioassay/1918958
Anticancer human tumor NCI-H211 cell line growth inhibition [LSD1]	Combinations_Synergy_Screen_LSD1	CSSLSD1_27	1,918,940	https://pubchem.ncbi.nlm.nih.gov/bioassay/1918940
Anticancer human tumor OVCAR-5 cell line growth inhibition	Combinations_Synergy_Screen_LSD1	CSSLSD1_28	1,918,956	https://pubchem.ncbi.nlm.nih.gov/bioassay/1918956
Anticancer human tumor SW 1271 cell line growth inhibition [LSD1]	Combinations_Synergy_Screen_LSD1	CSSLSD1_29	1,918,941	https://pubchem.ncbi.nlm.nih.gov/bioassay/1918941
Anticancer human tumor 156681-154-R-J1-PDC cell line growth inhibition [CLK]	Combinations_Synergy_Screen_CLK	CLK_01	2,060,627	https://pubchem.ncbi.nlm.nih.gov/bioassay/2060627
Anticancer human tumor 168753-222-R-J1-PDC cell line growth inhibition [CLK]	Combinations_Synergy_Screen_CLK	CLK_02	2,060,626	https://pubchem.ncbi.nlm.nih.gov/bioassay/2060626
Anticancer human tumor 186277-243-T-J2-PDC cell line growth inhibition [CLK]	Combinations_Synergy_Screen_CLK	CLK_03	2,060,625	https://pubchem.ncbi.nlm.nih.gov/bioassay/2060625
Anticancer human tumor 217524-143-R1-J4-PDC cell line growth inhibition [CLK]	Combinations_Synergy_Screen_CLK	CLK_04	2,060,624	https://pubchem.ncbi.nlm.nih.gov/bioassay/2060624
Anticancer human tumor 227483-062-R1-J1-PDC cell line growth inhibition [CLK]	Combinations_Synergy_Screen_CLK	CLK_05	2,060,623	https://pubchem.ncbi.nlm.nih.gov/bioassay/2060623
Anticancer human tumor 233499-124-R-J3-PDC cell line growth inhibition [CLK]	Combinations_Synergy_Screen_CLK	CLK_06	2,060,613	https://pubchem.ncbi.nlm.nih.gov/bioassay/2060613
Anticancer human tumor 242566-281-R-J2-PDC cell line growth inhibition [CLK]	Combinations_Synergy_Screen_CLK	CLK_07	2,060,622	https://pubchem.ncbi.nlm.nih.gov/bioassay/2060622
Anticancer human tumor 323965-272-R-J2-PDC cell line growth inhibition [CLK]	Combinations_Synergy_Screen_CLK	CLK_08	2,060,619	https://pubchem.ncbi.nlm.nih.gov/bioassay/2060619
Anticancer human tumor 324938-238-R-J1-PDC cell line growth inhibition [CLK]	Combinations_Synergy_Screen_CLK	CLK_09	2,060,621	https://pubchem.ncbi.nlm.nih.gov/bioassay/2060621
Anticancer human tumor 328373-195-R-J1-PDC cell line growth inhibition [CLK]	Combinations_Synergy_Screen_CLK	CLK_10	2,060,620	https://pubchem.ncbi.nlm.nih.gov/bioassay/2060620
Anticancer human tumor 377384-186-R-J1-PDC cell line growth inhibition [CLK]	Combinations_Synergy_Screen_CLK	CLK_11	2,060,616	https://pubchem.ncbi.nlm.nih.gov/bioassay/2060616
Anticancer human tumor 379773-124-R-J2-PDC cell line growth inhibition [CLK]	Combinations_Synergy_Screen_CLK	CLK_12	2,060,604	https://pubchem.ncbi.nlm.nih.gov/bioassay/2060604
Anticancer human tumor 422866-222-R5-J1-PDC cell line growth inhibition [CLK]	Combinations_Synergy_Screen_CLK	CLK_13	2,060,603	https://pubchem.ncbi.nlm.nih.gov/bioassay/2060603
Anticancer human tumor 454973-116-R2-J3-PDC cell line growth inhibition [CLK]	Combinations_Synergy_Screen_CLK	CLK_14	2,060,618	https://pubchem.ncbi.nlm.nih.gov/bioassay/2060618
Anticancer human tumor 485368-065-R4-J2-PDC cell line growth inhibition [CLK]	Combinations_Synergy_Screen_CLK	CLK_15	2,060,617	https://pubchem.ncbi.nlm.nih.gov/bioassay/2060617
Anticancer human tumor 496974-208-R-J2-PDC cell line growth inhibition [CLK]	Combinations_Synergy_Screen_CLK	CLK_16	2,060,615	https://pubchem.ncbi.nlm.nih.gov/bioassay/2060615
Anticancer human tumor 565232-114-T-J1-PDC cell line growth inhibition [CLK]	Combinations_Synergy_Screen_CLK	CLK_17	2,060,612	https://pubchem.ncbi.nlm.nih.gov/bioassay/2060612
Anticancer human tumor 598228-144-R-J1-PDC cell line growth inhibition [CLK]	Combinations_Synergy_Screen_CLK	CLK_18	2,060,614	https://pubchem.ncbi.nlm.nih.gov/bioassay/2060614
Anticancer human tumor 616215-338-R-J1-PDC cell line growth inhibition [CLK]	Combinations_Synergy_Screen_CLK	CLK_19	2,060,610	https://pubchem.ncbi.nlm.nih.gov/bioassay/2060610
Anticancer human tumor 628569-122-R-J1-PDC cell line growth inhibition [CLK]	Combinations_Synergy_Screen_CLK	CLK_20	2,060,611	https://pubchem.ncbi.nlm.nih.gov/bioassay/2060611
Anticancer human tumor 648629-189-R-J1-PDC cell line growth inhibition [CLK]	Combinations_Synergy_Screen_CLK	CLK_21	2,060,602	https://pubchem.ncbi.nlm.nih.gov/bioassay/2060602
Anticancer human tumor 817829-284-R-J1-PDC cell line growth inhibition [CLK]	Combinations_Synergy_Screen_CLK	CLK_22	2,060,599	https://pubchem.ncbi.nlm.nih.gov/bioassay/2060599
Anticancer human tumor 855422-203-R-J1-PDC cell line growth inhibition [CLK]	Combinations_Synergy_Screen_CLK	CLK_23	2,060,609	https://pubchem.ncbi.nlm.nih.gov/bioassay/2060609
Anticancer human tumor 874868-142-R-J2-PDC cell line growth inhibition [CLK]	Combinations_Synergy_Screen_CLK	CLK_24	2,060,601	https://pubchem.ncbi.nlm.nih.gov/bioassay/2060601
Anticancer human tumor 876135-273-R-J2-PDC cell line growth inhibition [CLK]	Combinations_Synergy_Screen_CLK	CLK_25	2,060,608	https://pubchem.ncbi.nlm.nih.gov/bioassay/2060608
Anticancer human tumor 876862-298-R-J1-PDC cell line growth inhibition [CLK]	Combinations_Synergy_Screen_CLK	CLK_26	2,060,607	https://pubchem.ncbi.nlm.nih.gov/bioassay/2060607
Anticancer human tumor 922993-354-T-J3-PDC cell line growth inhibition [CLK]	Combinations_Synergy_Screen_CLK	CLK_27	2,060,606	https://pubchem.ncbi.nlm.nih.gov/bioassay/2060606
Anticancer human tumor 947758-054-R-J2-PDC cell line growth inhibition [CLK]	Combinations_Synergy_Screen_CLK	CLK_28	2,060,605	https://pubchem.ncbi.nlm.nih.gov/bioassay/2060605
Anticancer human tumor 996289-038-R-J1-PDC cell line growth inhibition [CLK]	Combinations_Synergy_Screen_CLK	CLK_29	2,060,600	https://pubchem.ncbi.nlm.nih.gov/bioassay/2060600

## Discussion

Most genes with multiple introns and exons undergo alternative splicing to generate multiple mRNAs which are then translated into the diversity of proteins making up cellular proteins required for differentiation, development, and cell death in a process performed by spliceosomes, highly complex structures made up of approximately 300 proteins and RNA [44–47]. The Cancer Genome Atlas (TCGA) indicates that many solid tumors and hematologic malignancies deregulate DYRK1A. The pan-CLK/DYRK inhibitor cirtuvivint can cause programmed cell death at concentrations which inhibit the accumulation of phosphorylated SR proteins and alter splicing decreasing cellular proliferation in hematologic PDXs [45]. Cirtuvivint inhibits spliceosome associated CLK kinases especially SRSF5/6, thus, providing indirect inhibition of Wnt signaling resulting in antitumor activity [46]. Approximately 90% of colorectal cancers have mutations in the Wnt/β-catenin signaling pathway and aberrant Wnt signaling often occurs in gastric, pancreatic, breast and other cancers [46]. CC-671 is a potent and selective inhibitor of both TTK (human protein kinase monopolar spindle 1 [hMps1]) and CDC like kinase 2 (CLK2). A protein docking analysis indicated that CC-671 has high binding affinity to the drug-binding site of ABCG2 [47]. While dysregulation and alteration in pre-mRNA splicing is a recognized therapeutic target in hematologic malignancies, targeting pre-mRNA splicing has only been pursued recently for solid tumors. Pre-mRNA splicing modulation followed by PARP inhibition or chemotherapy in BRCA-mutant breast and ovarian cancers characterized by a “BRCA-ness” phenotype of dysfunctional homologous DNA repair [48].

Spliceosome-associated SR protein kinases SRPKs, CLKs, and NEK2 are altered in many cancers [49]. The CLK kinase family which includes CLK1-4, in conjunction with SRPK kinases adjust phosphorylation of SR proteins to modulate alternative splicing. CLK kinase activity changes are associated with cancer development and progression. Phosphorylation of SR proteins impact their subcellular localization, association with the spliceosome complex, and splicing activity [49]. SRSF5 and SRSF3 were reported to be overexpressed in oral squamous cell carcinoma (OSCC), and necessary for OSCC cell proliferation, cell cycle progression, and in vivo tumor formation. SRSF5-7 were found to be upregulated in small cell lung cancer (SCLC) and NSCLC tissues, and knockdown of SRSF5-7 in SCLC cell lines showed a significant decrease in proliferation [49].

Epigenetic mechanisms control gene expression patterns without change in DNA sequence. Histones, DNA binding proteins involved in regulation of nucleosome function, are subject to methylation, acetylation, phosphorylation, and ubiquitination. The methylation and demethylation of lysine residues on histone tails are post-translational protein modifications which control gene expression. The KDM (K = lysine) demethylase gene family includes 20 KDMs. KDM1s utilize a flavin adenine dinucleotide cofactor to demethylate methylated lysine substrates. KDM1A regulates many aspects of cell biology including self-renewal, differentiation, and stem cell pluripotency [50]. The protein encoded by the KDM1A gene is LSD1 which removes mono- and dimethyl groups from histone H34K and other chromatin-associated proteins. Two ways epigenetic therapies act as anticancer therapies are repression of oncogene function or activation of tumor-suppressor genes [51]. LSD1 is overexpressed in many proliferative diseases including hematological, lung, breast, and prostate cancers. Iadademstat, an irreversible LSD1 inhibitor, is in clinical development [52–54]. Several LSD1 inhibitors have completed Phase 1 clinical trial and have moved on to Phase 2 studies [55, 56]. Pevonedistat interferes with the function of the proteasome pathway blocking NAE. The PDMR 292921-168-R-J2 pancreatic carcinoma mct-spheroids was most responsive to pevonedistat reaching 2-logs of cytotoxicity and the K-562 leukemia, the PDMR 521955-158-R-J5 pancreatic carcinoma and the ovarian carcinoma lines OVCAR-5 and NCI/ADR-RES mct-spheroids reached 1-log of cytotoxicity upon exposure to pevonedistat for 7 days. The combination of iadademstat and pevonedistat resulted in increased cytotoxicity in the K-562 leukemia and the PDMR 556581-035-R-J1 ovarian carcinoma compared with single agent pevonedistat (Fig. 7). TAK-243 was markedly cytotoxic as a single agent in the K-562 leukemia, in 2 of 3 PDMR pancreatic carcinomas, 292921-168-R-J2 and 521955-158-R-J5, and the PDMR 556581-035-R-J1 ovarian carcinoma resulting in 3-logs of cytotoxicity at the highest concentration (0.3 µM) tested. There was little increase in response with iadademstat and TAK-243.

A first-in-human phase 1 iadademstat clinical study was conducted in relapsed refractory acute leukemia enriched with MLL/KMT2A-rearranged acute myeloid leukemia patients with most having MLL-translocation disease. The pharmacokinetic data indicate that the iadademstat clinical Cmax concentration was 0.116–0.182 nM, a concentration below the concentration range of 0.1–10 µM in the current study [57, 58]. Some patients with a molecular response to treatment with iadademstat showed blast cell differentiation, but no clinical responses were seen. In the current screen of 29 cell lines and 25 drugs and investigational agents indicate that response is highly cell line dependent. Although genetic marker(s) that might correlate with response to the combination regimens were sought no clear marker(s) emerged.

## Data Availability

All data are available in PubChem as shown in the manuscript.
